# A Novel Time–Frequency Similarity Method for P-Wave First-Motion Polarity Detection

**DOI:** 10.3390/s25134157

**Published:** 2025-07-03

**Authors:** Yanji Yao, Xin Xu, Jing Wang, Lintao Liu, Zifei Ma

**Affiliations:** 1 School of Water Conservancy, Yunnan Agricultural University, Kunming 650201, China; yaoyanji@asch.whigg.ac.cn (Y.Y.); wj@ynau.edu.cn (J.W.); 2 State Key Laboratory of Precision Geodesy, Innovation Academy for Precision Measurement Science and Technology, Chinese Academy of Sciences, Wuhan 430077, China; llt@asch.whigg.ac.cn; 3 Yunnan Earthquake Agency, Kunming 650224, China; xuxin3064@126.com

**Keywords:** normal time–frequency transform, time–frequency similarity coefficient, P-wave first-motion polarity, seismic noise

## Abstract

P-wave first-motion polarity is a critical parameter for determining earthquake focal mechanisms. Extracting relative P-wave arrival times and polarity information using waveform cross-correlation techniques can enhance the accuracy of earthquake location and focal mechanism inversion. However, seismic noise severely hampers the reliable detection of P-wave onsets and their first-motion polarities. To address this issue, we propose a noise-resistant polarity detection method based on the normal time–frequency transform (NTFT), termed the time–frequency similarity coefficient (TFSC). The TFSC method computes relative delays and similarity coefficients by calculating the real part of the NTFT coefficients between two seismic signals. We validated the proposed approach using both synthetic and real earthquake data. Without any filtering or preprocessing, the TFSC method demonstrated significantly greater robustness and reliability compared to the conventional time-domain normalized cross-correlation (NCC) method. These results indicate that the TFSC method has strong potential for practical application and provides a novel perspective for accurate detection of P-wave first-motion polarity in noisy seismic environments.

## 1. Introduction

Determining earthquake hypocenters and focal mechanisms requires precisely measured P-wave arrival times and first-motion polarities [1,2,3]. The identification of P-wave first-motion polarity is a critical research focus in seismology, with significant applications in focal mechanism inversion and underground nuclear explosion monitoring. The inversion of focal mechanisms using P-wave first-motion polarity is simple and efficient, and it has been widely employed for determining focal mechanisms of small to moderate earthquakes. Wu et al. [4] proposed a workflow that incorporates machine learning-based phase picking and P-wave first-motion polarity picking, followed by rapid phase association, precise earthquake location, and template matching for detecting small earthquakes to enhance the completeness of the earthquake catalog. Nakamura [5] proposed an automated approach that band-pass-filters S-wave displacement data with a Bessel filter, detects P- and S-wave onset polarities via an autoregressive model refined by an AR filter, and applies a grid-search inversion to obtain a reliable focal-mechanism solution. Ross et al. [3] trained a deep learning model using 18.2 million manually picked seismic waveforms from southern California, and effectively used the model to perform P-wave arrival picking and first-motion polarity determination. Chen et al. [6] proposed a deep learning method (EQPolarity) for determining the P-wave first-motion polarity using the vertical-component seismic waveforms. Li et al. [7] proposed an automatic workflow, FocMech-Flow (Focal Mechanism-Flow), for identifying P-wave first-motion polarity and focal mechanism inversion with deep learning and applied it to the 2021 Yangbi earthquake sequence. Zhao et al. [8] proposed a deep learning-based method for automatic first-motion polarity identification called “DiTingMotion” and combined it with the HASH method to develop an automated focal mechanism inversion workflow. This workflow utilizes the first-motion polarities identified by DiTingMotion and was applied to the 2019 M6.4 Ridgecrest earthquake sequence for performance evaluation. A large number of first-motion polarity records from small earthquakes, including those that cannot independently constrain focal mechanisms, can still be effectively used to infer stress field directions [9]. Uchide [10,11] employed deep learning to automatically pick P-wav first-motion polarities and inverted the focal mechanisms for over 110,000 inland earthquakes in Japan. Uchide et al. [12] used a deep learning model to determine first-motion polarities for over 220,000 microearthquakes, leading to a more accurate and comprehensive crustal stress map of Japan. Pei and Zhou [9] proposed a novel high-precision probabilistic approach for automatic P-wave onset and polarity detection (the POI method), based on which they developed a fully automated inversion workflow for small earthquake focal mechanisms and applied it to the Xiaojiang Fault zone in Yunnan, China. The primary advantage of using P-wave first-motion polarity for focal mechanism inversion of small earthquakes lies in its simplicity and efficiency. However, this method may struggle to ensure reliability when data quality is uneven or data volume is insufficient.

Traditionally, P-wave first-motion polarities were manually picked, but with the advancement of artificial intelligence, automatic detection methods have reduced the biases associated with manual operations. Pei and Zhou [9] pointed out that although AI-based methods can achieve high accuracy, they require a large amount of labeled sample data to train the models. Therefore, transferring and applying these methods across different regions and datasets poses certain challenges and can be time-consuming. Nevertheless, the picking accuracy still needs improvement when applied to different regions [13]. Accurate identification of P-wave first-motion polarity is essential for constraining focal mechanism parameters and improving the reliability of stress field interpretations. The cross-correlation (CC) is an effective tool for measuring waveform similarity and has been widely used in seismology, including for full waveform inversion [14], the detection of random noise signals [15], the clustering of similar events [16], the correlation analysis of different surface wave components [17], and quasi-Love wave observation [18,19]. The normalized cross-correlation coefficient (NCC) is commonly used to evaluate waveform similarity or detect known events within noisy data [19]. While NCC performs well for high-SNR waveforms, its performance degrades in the presence of noise, potentially leading to false detections. To enhance robustness, the generalized cross-correlation (GCC) method was introduced [20], incorporating pre-filtering and noise suppression prior to correlation computation. However, traditional filtering methods for seismic signals can introduce phase distortion, energy leakage, or artifacts [21]. In the GCC method, the time delay is estimated by applying a Fast Fourier Transform (FFT) to the signals, weighting the cross-power spectrum in the frequency domain, and then performing an inverse transform. However, as seismic waves are non-stationary and often contain non-Gaussian noise, and since the traditional Fourier transform does not provide time-localization information, the FFT-based GCC method is not well suited for such signals [19]. Compared with time-domain or frequency-domain methods alone, time–frequency analysis techniques can better characterize signal structures and suppress noise interference. Wavelet transform is suitable for analyzing non-stationary signals; for instance, wavelet and Hilbert–Huang transforms have recently been integrated into GCC to improve performance [22,23,24]. Nonetheless, the performance of wavelet-based GCC heavily depends on the choice of wavelet basis. Moreover, wavelet transforms defined in the L2(R) space can suffer from frequency shifting [25], while the S-transform (ST) may provide inaccurate frequency resolution at high frequencies [26], potentially leading to phase shifts and errors in determining seismic parameters. The normal time–frequency transform (NTFT) is a relatively recent wavelet-based method using the L1-norm, is free from traditional admissibility conditions [25], and is ideal for analyzing harmonic and quasi-harmonic signals. The NTFT is the inheritance and development of wavelet transform and S-transform, and it can represent the real-time characteristics of the signal unbiasedly, improve the problems of pseudo-frequency, frequency shifts or inaccuracy, and can retain the information of the original signal to the greatest extent [25]. A novel time–frequency domain cross-correlation method, referred to as the time–frequency similarity coefficient (TFSC) method, is proposed for detecting P-wave first-motion polarity. In this method, seismic signals are first transformed using the NTFT, and similarity analysis is then performed based on the normal time–frequency transform coefficients. Accurately detecting P-wave first-motion polarity without the need for filtering or other preprocessing steps is the motivation of this paper.

## 2. Methods

### 2.1. Time–Frequency Similarity Coefficient (TFSC) Theory

The TFSC method is proposed based on the NTFT in the time–frequency domain. It evaluates the similarity between two signals by computing the real part of the NTFT coefficients, with a value range of [−1, 1]. This coefficient similarity can be used for time delay estimation or signal detection. For two time-domain signals *f*1(t) and *f*2(t), the TFSC is defined as follows:

(1) ρ(l, s)=∬S+lRe(Ψf1(τ, ῶ))Re(Ψf2(τ+s, ῶ))dτdῶ∬S+l(ReΨf1(τ, ῶ))2dτdῶ∬S+l(ReΨf2(τ+s, ῶ))2dτdῶ,

where *l* ∈ *R*, and Ψ denotes the NTFT, as defined in Equation (3). Here, *τ* and ῶ represent the instantaneous time and instantaneous frequency, respectively. Re denotes the real part operator. *s* represents the time delay, where *s* ∈ Δ, and Δ denotes the time delay domain. *S* indicates the region of interest in the time–frequency domain, and *S* + *l* represents the target region *S* is shifted by *l* time units.

The NTFT serves as a basic framework for normal time–frequency transformations. By assigning different values or expressions to *μ*(ῶ) and the window function *w*(*t*) in the kernel function defined in Equation (2), various types of time–frequency transforms can be derived.

A typical NTFT kernel function can be defined as follows:

(2) ψ(t,ῶ)=|μ(ῶ)|w(μ(ῶ)t)exp (jῶt),(μ(ῶ)∈R)≠0

where “||” is the modulus operator. The local time function *w*(*t*) adopts a normal Gaussian window. The *μ*(*ῶ*) is the time–frequency resolution adaptor function and can be almost any mathematical expression or constant except zero. For example, when *μ*(*ῶ*) = 1, the NTFT reduces to the normal Gabor transform.

In this study, because seismic signals are broadband signals, the kernel function of the NTFT is constructed under the following conditions:

(3) $$\left\{\begin{array}{l} \mu (ῶ)=ῶ\\ w(t)=\frac{1}{\sqrt{2\pi }\sigma }exp(-\frac{t^{2}}{2\sigma^{2}}) \end{array}\right.$$

The expression for the NTFT is as follows:

(4) $$\Psi f\left(\tau ,ῶ\right)=\frac{\left|ῶ\right|}{\sqrt{2\pi }\sigma }\int_{-\infty }^{+\infty }f\left(t\right)exp\;(-\frac{{ῶ}^{2}{\left(t-\tau \right)}^{2}}{2\sigma^{2}}+jῶ(\tau -t))dt\;$$

where the kernel function of the NTFT is shown below.

(5) $$\psi \left(t,ῶ\right)=\frac{\left|ῶ\right|}{\sqrt{2\pi }\sigma }exp\;(-\frac{{\left(ῶt\right)}^{2}}{2\sigma^{2}}+jῶt)$$

In addition, we found that the TFSC can also be defined as follows:

(6) $$\rho (l,\;s)=\frac{\left|\iint_{S+l}\Psi f_{1}(\tau ,\;ῶ)\overset{-}{\Psi f_{2}(\tau +s,\;ῶ)}d\tau dῶ\right|}{\sqrt{\iint_{S+l}|\Psi f_{1}(\tau ,\;ῶ){|}^{2}d\tau dῶ\iint_{S+l}|\Psi f_{2}(\tau +s,\;ῶ){|}^{2}d\tau dῶ}}$$

The similarity between two signals can also be calculated based on the modulus of the normal time–frequency transform coefficients, with the similarity value in the range [0, 1]. Additionally, for seismic signal analysis, the time delay between signals *f*_1_(*t*) and *f*_2_(*t*) can be determined by computing the similarity coefficient using Equation (6), taking the modulus of the result, and selecting the time corresponding to the maximum similarity coefficient, as shown in Equation (7).

(7) $$\left|\rho (l,\;s^{\prime })\right|=\underset{s\in \Delta }{\max}\left(\left|\rho (l,\;s)\right|\right)$$

Here, ‘||’ denotes the modulus operation, and max indicates the selection of the maximum value. The value s′ can be interpreted as the time delay of the target signal segment in *f*_2_(*t*) relative to the corresponding position in *f*_1_(*t*). When the template signal and the target signal segment are positively correlated in phase, the time–frequency similarity coefficient is positive. Conversely, when the template and target signal segments are correlated with opposite phases, the coefficient is negative.

### 2.2. Comparison with the NCC Method

The normalized cross-correlation coefficient (NCC) is defined as follows:

(8) $$\mathrm{NCC}(\tau )=\frac{\sum_{n=0}^{N-1}f_{1}(n)f_{2}(n+\tau )}{\sqrt{\sum_{n=0}^{N-1}{f_{1}}^{2}(n)\sum_{n=0}^{N-1}{f_{2}}^{2}(n)}}$$

where *τ* represents the time delay between two discrete signals *f*_1_(*n*) and *f*_2_(*n*). The NCC is commonly used to evaluate the correlation between two signals and to estimate the time delay [19].

**Figure 1:**
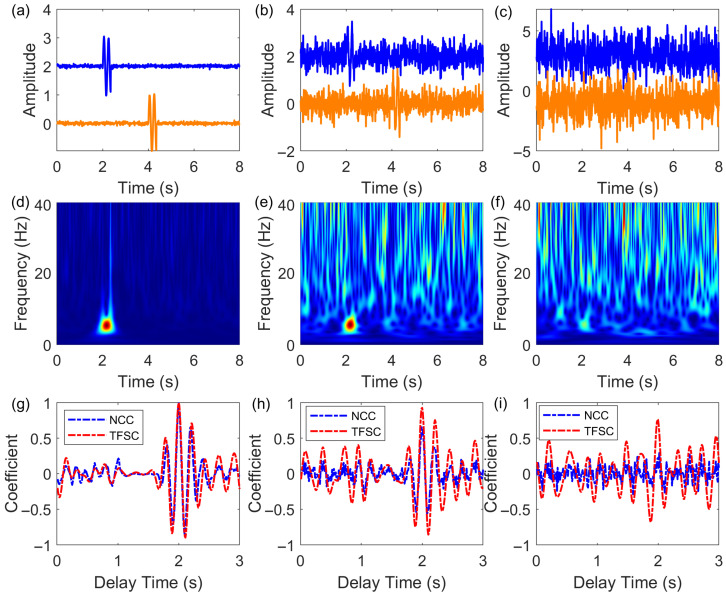
Comparison of the NCC method and TFSC method under different noise levels. (**a**–**c**) Simulated template signals (blue lines) and time-shifted target signals (orange lines) at SNR levels of 30 dB, 10 dB, and 0 dB, respectively. (**d**–**f**) Normal time–frequency amplitude spectra of the template signals (blue lines in **a**–**c**) at different SNR levels. (**g**–**i**) Comparison of similarity coefficients and estimated delays calculated by the NCC and TFSC methods under different SNR conditions.

To verify the effectiveness of the TFSC method, we compared the time delay estimation performance of the TFSC and NCC methods using simulated signals. Gaussian white noise at different levels (30 dB, 10 dB, and 0 dB) was added to the simulated signals for comparison, as shown in Figure 1. Two identical simulated signals were used, with a theoretical time delay of 2 s. The time–frequency characteristics of the signals after noise injection are shown in Figure 1d–f. In a high signal-to-noise ratio (SNR = 30 dB) scenario, both the NCC and TFSC methods produced consistent results in terms of estimated delay and similarity coefficients (Figure 1a,g). When the noise level increased to SNR = 10 dB, the performances of the NCC and TFSC methods show a distinct difference. The delay calculated by the NCC method is 1.99 s with a similarity coefficient of 0.71. Under the same noise conditions, the delay computed by the TFSC method is 2 s, with a time–frequency similarity coefficient of 0.96 (Figure 1b,h). Under strong noise conditions with a SNR of 0 dB, the similarity coefficient calculated by the NCC method dropped below 0.5, making it ineffective for delay estimation. In contrast, the TFSC method yielded a similarity coefficient of 0.77 and a delay of 1.99 s under the same noise level (Figure 1c,i). Compared with the NCC method, the TFSC method exhibits more distinct response characteristics. The TFSC method calculates the similarity between two signals in the time–frequency domain based on their time–frequency features. Therefore, under noisy conditions, the TFSC method demonstrates greater robustness.

## 3. Results

### 3.1. Real Data

**Figure 2:**
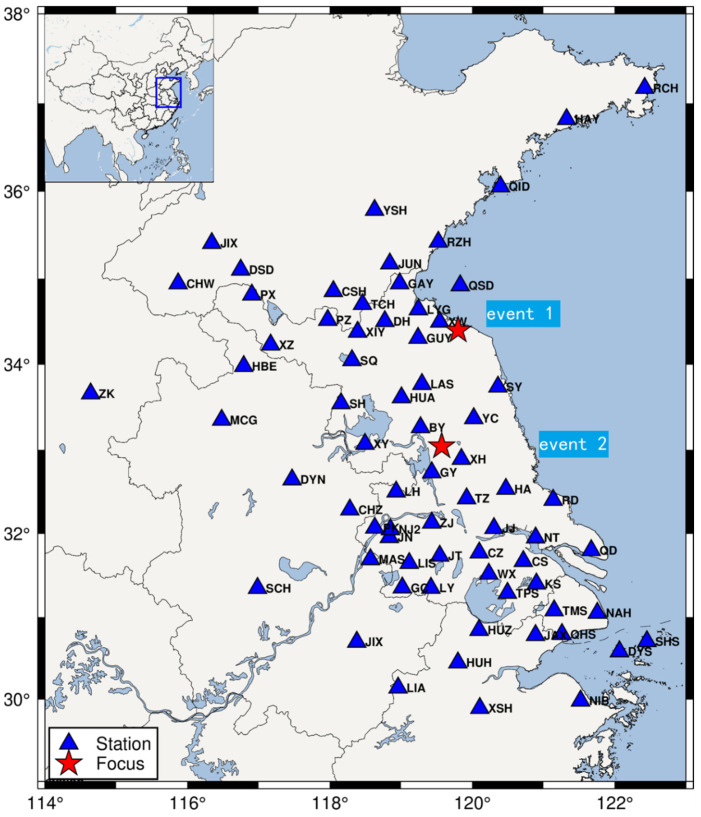
Distribution of seismic stations in the study area.

To verify the accuracy and reliability of the TFSC method, we collected waveform records from 70 seismic stations across different seismic networks for the P-wave first-motion polarity extraction study, as shown in Figure 2. Seismic Event 1 is a magnitude 3.5 earthquake that occurred on 19 January 2013, in Xiangshui County, Jiangsu Province (epicenter: 34.4° N, 119.8° E), with a focal depth of 5 km. The average distance between stations near Seismic Event 1 is 52.21 km, and the closest station is 26.05 km from the event. Seismic waveform records are subject to varying levels of environmental noise interference. Therefore, the waveform data from stations closest to the seismic event are more reliable for constraining the epicenter location. Seismic Event 2 is a magnitude 4.9 earthquake that occurred on 20 July 2012, in Gaoyou, Jiangsu Province (epicenter: 33.04° N, 119.57° E), with a focal depth of 15 km. This event is associated with a branch fault of the Tan-Lu fault zone (not the main fault). The average distance between stations near Seismic Event 2 is 61.84 km, with the closest station 30.83 km from the event. According to the Global CMT solution, the seismic source mechanism of Event 2 has a strike of 22°, a dip angle of 73°, and a rake angle of 177° (https://www.globalcmt.org/CMTsearch.html, accessed on 30 May 2025).

### 3.2. Robustness Analysis of P-Wave First-Motion Polarity Picking

To reduce false positives caused by noise during P-wave triggering at seismic stations, a similarity coefficient threshold of ±0.5 was applied in the TFSC method. This threshold was empirically determined based on 70 sets of experimental samples, and the recognition performance was evaluated under different threshold settings. To verify the reliability of the proposed method, a cross-validation was carried out by comparing the identified results with manually picked P-wave first-motion polarities. The results show that when the threshold was set to 0.4, a false positive rate of 8.57% was observed; when set to 0.5, the false positive rate dropped to 2.86% with no significant false negatives detected; and when increased to 0.6, the false positive rate was further reduced to 1.43%, but the false negative rate rose to 18.57%. Considering both recognition accuracy and error control, a threshold of ±0.5 is regarded as a relatively reasonable and balanced choice. If the TFSC coefficient lies within the range of [–0.5, +0.5], the two signals are considered dissimilar. We analyzed the waveforms from all stations triggered by the earthquake using the TFSC method and compared the results with those obtained using the NCC method. It is worth noting that, in theory, when the hypocenter and two seismic stations lie on a straight line, the difference in P-wave arrival times between the two stations corresponds to the maximum delay. Thus, the inter-station distance can be used to pre-estimate the sliding time window used during waveform matching.

**Figure 3:**
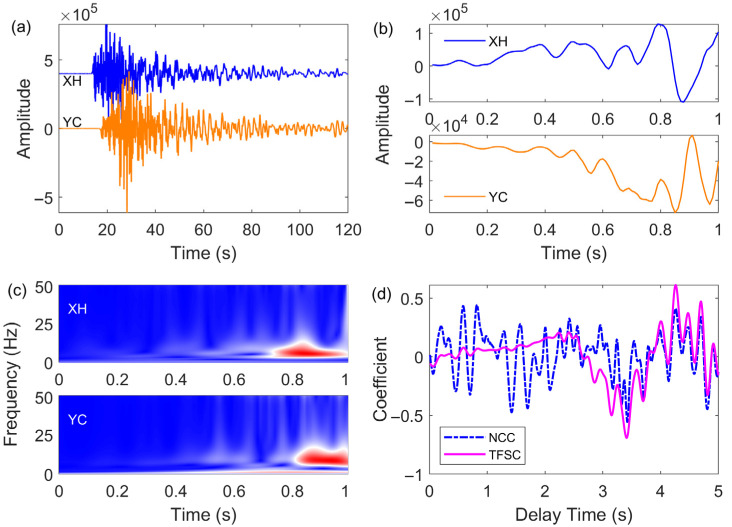
Comparison of the TFSC and NCC methods. (**a**) Seismic waveforms from two stations. (**b**) Template signal and target signal to be matched using the NCC method. (**c**) Template signal and target signal to be matched using the TFSC method. (**d**) Similarity coefficients and delays calculated using the NCC and TFSC methods.

For a fair comparison, both the TFSC and NCC methods adopted the same sliding window parameter. In both approaches, a 1 s waveform segment following the P-wave arrival was selected as the template signal. The maximum absolute value of the similarity coefficient indicates the best match between two signals, and the corresponding time delay represents the P-wave arrival time difference. Figure 3 presents a comparison of the delay and similarity coefficients computed by TFSC and NCC for the earthquake signals. The waveform segment within 1 s after the P-wave arrival at station XH was selected as the template (Figure 3a) and matched against the signal at station YC. The waveform comparison between the NCC template and target signal is shown in Figure 3b. The TFSC method, in contrast, uses NTFT to transform the signals, and matching is performed using the corresponding time–frequency spectral coefficients (Figure 3c). Figure 3d shows the similarity coefficient series obtained from both methods. When the P-wave first-motion polarities of the template and target signals are opposite, the resulting similarity coefficient is negative. The manually determined P-wave arrival time difference between XH and YC is 3.38 s. The NCC method yields a maximum absolute similarity coefficient of 0.556 (with opposite polarity, −0.556) and a delay of 3.42 s. The TFSC method gives a maximum absolute similarity coefficient of 0.694 (with opposite polarity, −0.694) and a delay of 3.41 s. The peak feature in the TFSC result is more distinct and easier to identify. Compared to the time-domain NCC cross-correlation method, the time–frequency domain TFSC method provides more stable and accurate matching of the target signal.

**Figure 4:**
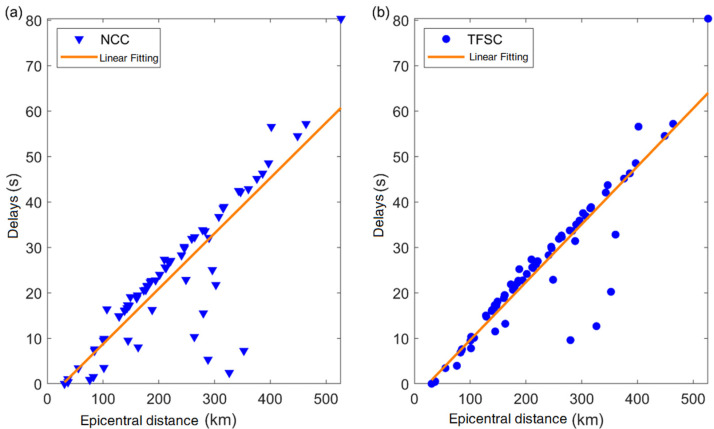
Comparison of time delays calculated by the NCC and TFSC methods. (**a**) Time delays estimated by the NCC method plotted against epicentral distance. (**b**) Time delays estimated by the TFSC method plotted against epicentral distance, showing fewer outliers.

**Figure 5:**
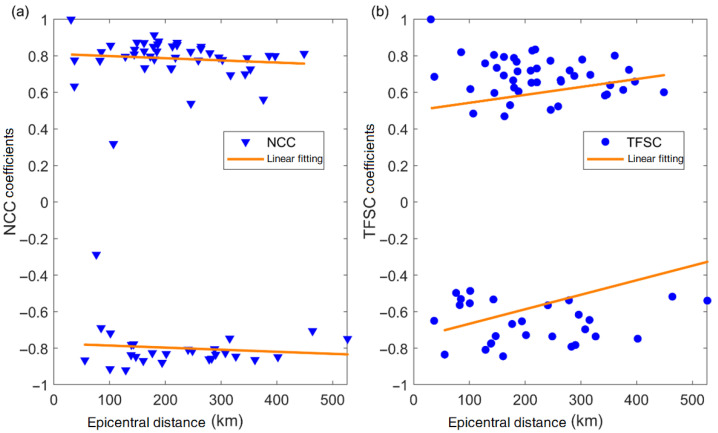
Comparison of similarity coefficients calculated using the NCC and TFSC methods. (**a**) Similarity coefficients calculated by the NCC method versus epicentral distance. (**b**) Similarity coefficients calculated by the TFSC method versus epicentral distance. The TFSC method is more sensitive to structural variations in the signal.

To verify the reliability of the proposed TFSC method, we applied both the TFSC and NCC methods to the Ms4.9 earthquake in Gaoyou, Jiangsu Province. We performed a statistical analysis of the similarity coefficients and P-wave arrival time differences from 70 seismic stations. The accuracy of the P-wave arrival time difference was cross-validated by correlating it with the actual epicentral distance. We selected the waveform (1 s) from the first triggered station (XH) as the template signal and matched it against waveforms from the other stations, with a sliding step size of 0.01 s. As shown in Figure 4, we correlated the P-wave arrival time differences calculated by the two methods with the epicentral distance to assess their reliability. The results indicate that the arrival time differences computed by the NCC method exhibit larger deviations, whereas the TFSC method yields more stable results. Compared to the NCC method, the TFSC method demonstrates better noise resistance, with fewer outliers. Most of the calculated P-wave arrival times were consistent with the actual variations in epicentral distance. Figure 5 presents the statistical comparison of similarity coefficients and epicentral distances for both methods. The similarity coefficients obtained by the NCC method are relatively uniform with small variations, which may limit its ability to discriminate against interference signals. In comparison, the similarity values derived from the TFSC method are more dispersed and exhibit a broader range of variation, reflecting its greater sensitivity to signal structure differences caused by noise. To verify the reliability of the proposed method, a cross-validation was conducted between manually picked P-wave first-motion polarities and the detection results of the two methods using the same dataset. As shown in Table 1, the picking accuracy of P-wave first-motion polarity detection by the NCC method is 82.86%, while the proposed TFSC method achieves an accuracy of 91.43%. Additionally, variations in time delay and similarity coefficients further demonstrate the good robustness of the TFSC method in noisy environments.

**Figure 6:**
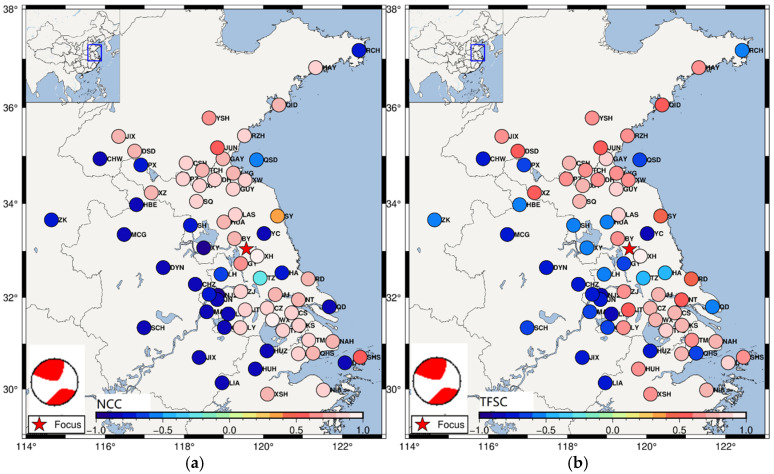
Comparison of coefficient distribution calculated by the NCC and TFSC methods. (**a**) Variation in the distribution of similarity coefficients calculated by the NCC method for signals from different stations. (**b**) Variation in the distribution of similarity coefficients calculated by the TFSC method for signals from different stations. The similarity coefficients calculated by the NCC method show relatively small variations and are more concentrated and uniform with respect to the station distribution. In contrast, the similarity coefficients calculated by the TFSC method exhibit a richer variation with respect to station positions. This indicates that the TFSC method more accurately reflects subtle changes in the signal components.

Furthermore, the variation in similarity coefficients calculated by the NCC and TFSC methods with the station distribution is analyzed, as shown in Figure 6. In the figure, positive values of similarity coefficients indicate positive P-wave first-motion polarity, while negative values indicate negative polarity. The Ms 4.9 earthquake in Gaoyou, Jiangsu Province, was a strike-slip event along the Chuhe Fault, with a strike orientation of either north-northeast or northwest. The Chuhe Fault, located near the epicenter, trends north-northeast and extends across Jiangsu and Anhui Provinces to the Yellow Sea. The region is seismically active, with significant tectonic movements caused by the ongoing compressional forces from the Tethys and enhanced Pacific tectonics. The eastern part of China exhibits NE-striking sinistral shear movements, particularly along the Tan-Lu fault zone [27]. As shown in the source sphere from the Global CMT solution in Figure 6, the P-wave first-motion polarity results calculated using both the NCC and TFSC methods show a quadrant distribution that is consistent with the strike-slip mechanism derived from the Global CMT solution. The negative and positive correlations calculated by the NCC method reflect the correlation between signals, with strong correlations and small variations in the coefficient values. The positive and negative coefficients calculated by the TFSC method reflect the similarity between signals, with a more diverse distribution of coefficient values. This indicates that the TFSC method is more sensitive to structural changes in the seismic signal.

## 4. Discussion

The accuracy of P-wave arrival time picking directly affects the determination of initial motion polarity, particularly when interference or high noise levels are present before the P-wave arrival. Such conditions can lead to incorrect determination of the initial motion polarity. During an earthquake, seismometers may be subject to human-induced noise, environmental noise, or instrument-related interference. These interference signals can result in incorrect P-wave picking, or even failure to detect the P-wave, leading to significant errors in epicenter location or an inability to determine the epicenter. To address this issue before locating the epicenter, interference signals are removed based on the TFSC similarity coefficient. Through extensive experiments and statistical analysis of the time–frequency similarity coefficients, the threshold for the TFSC method is set at ±0.5.

Assuming a constant seismic wave propagation velocity, the first three seismic stations that receive the P-wave are considered. The first station to receive the P-wave is denoted as Sta1, and the second as Sta2. According to the TFSC threshold, if the TFSC coefficient between Sta1 and Sta2 is greater than or equal to 0.5 or less than or equal to −0.5, indicating that the two waveforms are either highly positively correlated or highly negatively correlated, the P-wave arrival time difference is calculated using the TFSC method and used for epicenter location. If the coefficient falls into the ambiguous interval (0.4 < TFSC < 0.5 or −0.5 < TFSC < −0.4), a third station, Sta3, is introduced for further evaluation. If the TFSC coefficient between Sta1 and Sta3 is greater than or equal to 0.5 or less than or equal to −0.5, and Sta2 shows a significant difference from both, the signal from Sta2 is considered to be affected by interference. If all three TFSC coefficients fall within the non-similar range (i.e., |TFSC| ≤ 0.4), Sta3 is redefined as the new Sta1, and a new combination of stations, including Sta4 and Sta5, is constructed for the next round of discrimination. To prevent logical loops or instability caused by recursive redefinition of Sta1, the number of iterations is limited to two. If no valid result is obtained after two iterations, the current epicenter status is marked as “undetermined,” and further judgment is deferred until more waveform data become available. Additionally, to enhance the robustness and anti-interference capability of epicenter determination, the Short-Time Average/Long-Time Average (STA/LTA) method is employed for cross-validation to assist in the evaluation of waveform quality by the TFSC method. If a station is identified as anomalous by both the TFSC and STA/LTA indicators, the corresponding signal is considered to be interfered and is excluded, thereby improving the accuracy of epicenter localization.

**Figure 7:**
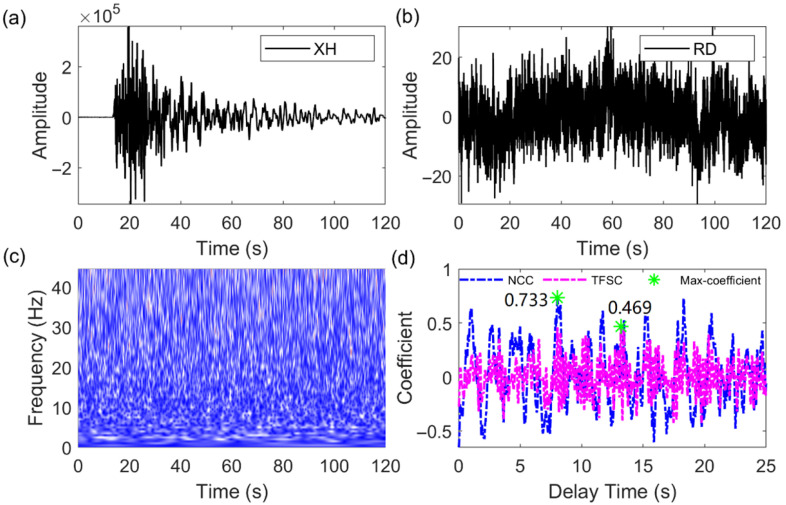
Strong noise interference signal identified by the NCC and TFSC methods. (**a**) Template signal. (**b**) Target signal to be matched. (**c**) NTFT time–frequency amplitude spectrum corresponding to (**b**), used to assess whether the signal contains earthquake characteristics. (**d**) The similarity coefficients and delay variations computed by the NCC and TFSC methods through (**a**,**b**). The green ‘*’ in the figure represents the maximum value of the absolute similarity coefficient, corresponding to the delay between the two signals.

**Figure 8:**
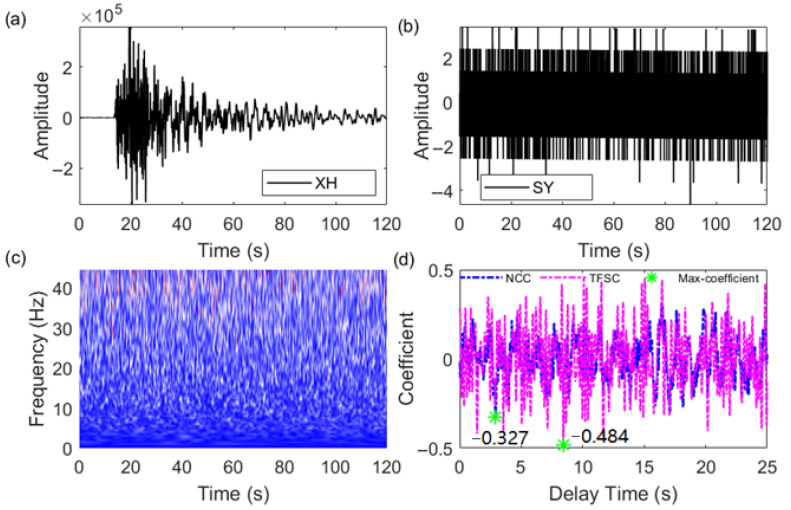
Instrument pulse interference signal discrimination by the NCC and TFSC methods. (**a**) Template signal. (**b**) Target signal to be matched. (**c**) NTFT time–frequency amplitude spectrum corresponding to (**b**), used to assess whether seismic signals are present. (**d**) Similarity coefficients and delay variations calculated by the NCC and TFSC methods through (**a**,**b**). The green ‘*’ in the figure indicates the maximum absolute value of the similarity coefficient, corresponding to the delay between the two signals.

Interference signals are identified based on the NCC and TFSC similarity coefficients and the set threshold. As shown in Figure 7b, the RD station is affected by environmental noise, making it impossible to determine if there is an earthquake signal. When matched with the waveform from the XH station (Figure 7a), the maximum correlation coefficient calculated by the NCC method is 0.733, and the maximum similarity coefficient calculated by the TFSC method is 0.469. Based on the set threshold, the TFSC method identifies the signal as interference. Further examination using amplitude features and the NTFT time–frequency spectrum helps filter out noise signals. The time–frequency spectrum does not show distinct earthquake signal features, as shown in Figure 7c. As shown in Figure 7d, the P-wave time differences calculated by the NCC and TFSC exhibit large deviations, making it difficult to determine the accuracy of the delay. Under strong noise interference, the similarity coefficients calculated by the TFSC method are consistently below the threshold, while those calculated by the NCC method are higher (>0.5). Additionally, for instrument pulse noise interference, as shown in Figure 8, both the NCC and TFSC methods calculate coefficients below the threshold, effectively identifying the pulse interference signal. While the TFSC method may not detect all interference signals with 100% accuracy, it significantly reduces certain interference signals, thereby enhancing the reliability of P-wave first-motion polarity detection and improving the accuracy of the source mechanism solution.

**Figure 9:**
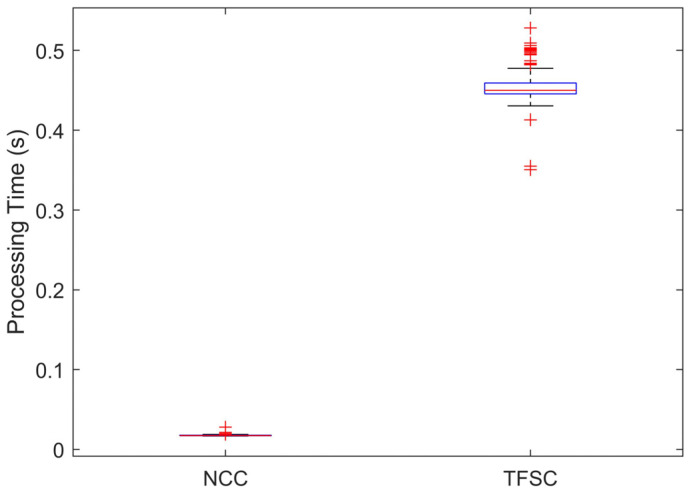
Comparison of computational time for the two algorithms based on real data testing.

Furthermore, the computational complexity of the proposed TFSC algorithm was compared with that of the NCC algorithm, and the comparison results are shown in Table 2. All experiments were conducted on a laptop equipped with an Intel Core i7-10750H CPU @ 2.60 GHz, 64 GB RAM, and an NVIDIA GeForce GTX 2060 GPU. We evaluated the processing time required by the TFSC and NCC methods on 210 seismic signals recorded from 70 seismic stations. As shown in Figure 9, the experimental results indicate that the TFSC method takes approximately 0.44 s on average to process a single seismic signal. Compared to the NCC algorithm, the improved accuracy of the TFSC method comes at the expense of reduced computational efficiency. According to the convolution theorem, there is still room for optimizing the computational complexity of the TFSC method. Optimizing the algorithmic structure or deploying the method in a higher-performance computing environment is expected to further reduce the required computation time.

## 5. Conclusions

The P-wave first-motion polarity is crucial for the inversion of earthquake source mechanisms and is widely used in the study of source mechanisms for medium to small earthquakes. However, the traditional time-domain NCC method is prone to false detections under noise interference, which adversely affects the accuracy of polarity identification. To improve the recognition performance of P-wave first-motion polarity in complex environments, the TFSC method was proposed, enabling accurate detection of P-wave first-motion polarity in the time–frequency domain. In this method, seismic signals are transformed using the NTFT, and the similarity is calculated based on the real part of the time–frequency coefficients. Compared with the traditional time-domain NCC method, the TFSC method was shown to possess stronger noise resistance and higher sensitivity to structural differences in signals, leading to a significant improvement in polarity recognition accuracy. In particular, better robustness and adaptability were demonstrated under complex noise conditions.

The main contributions of this study can be summarized as follows:

- A novel similarity measure in the time–frequency domain was introduced, by which accurate polarity detection is achieved without the need for preprocessing such as filtering.
- A threshold-based decision mechanism was constructed to balance false positives and false negatives, enhancing the detection accuracy of polarity under low signal-to-noise ratio conditions.
- Through experiments on real earthquake data, the TFSC method was validated to achieve a P-wave first-motion polarity detection accuracy of 91.43%, significantly outperforming the NCC method’s accuracy of 82.86%.

In addition, a more dispersed distribution of similarity coefficients was observed under noisy conditions by using the TFSC method, indicating greater sensitivity to signal variations caused by noise interference. These advantages indicate that the method is considered to have good application prospects in automatic polarity recognition in seismology and can be further extended to areas such as focal mechanism inversion and repeating earthquake identification. The applicability of this method in real-time seismic monitoring systems will be further investigated in future work.

## Figures and Tables

**Table 1:** Accuracy of P-wave first-motion polarity detection by two methods (referenced to manually picked results).

Methods	NCC	TFSC
Picking accuracy	82.86%	91.43%

**Table 2:** The transform domains and average execution time of the two algorithms.

Methods	NCC	TFSC
Transform domain	Time domain	Time–frequency domain
Execution time (seconds)	0.02	0.44

## Data Availability

The data presented in this study are available upon request from the corresponding author. The data are not publicly available due to privacy concerns.

## Abbreviations

The following abbreviations are used in this manuscript:

NTFT	Normal Time–Frequency Transform
TFSC	Time–Frequency Similarity Coefficient
NCC	Normalized Cross-Correlation
